# Problematic Smartphone Use and Quality of Life Among Greek Nursing Students: A Cross-Sectional Study

**DOI:** 10.3390/ijerph23070870

**Published:** 2026-07-03

**Authors:** Georgios Manomenidis, Vasiliki Georgousopoulou, Elena Vasileiou, Savvato Karavasileiadou, Nikoletta T. Karavasili, Stefanos Mavroudis, Eman Atef

**Affiliations:** 1 Department of Nursing, Democritus University of Thrace, 68100 Alexandroupolis, Greece; vageorgo@nurs.duth.gr (V.G.); nkaravas@nurs.duth.gr (N.T.K.); ste_mavroudis@yahoo.gr (S.M.); 2 Department of Occupational Health Services, University Hospital of Alexandroupolis, 68100 Alexandroupolis, Greece; occmed1@pgna.gr; 3 Department of Community and Psychiatric Mental Health Nursing, College of Nursing, Princess Nourah Bint Abdulrahman University, Riyadh 11671, Saudi Arabia; skaravasileiadou@pnu.edu.sa; 4 Faculty of Nursing, Helwan University, Cairo 11795, Egypt; eman_atef@nursing.helwan.edu.eg

**Keywords:** problematic smartphone use, nursing students, quality of life, Greek

## Abstract

**Highlights:**

**Public health relevance—How does this work relate to a public health issue?**

- Problematic smartphone use is an emerging public health concern among nursing students, with potential effects on well-being, daily functioning, and academic life.
- This study highlights the need to address digital overuse within health-profession education, particularly as smartphones are increasingly embedded in learning and clinical routines.

**Public health significance—Why is this work of significance to public health?**

- Nearly one in five nursing students screened positive for potential problematic smartphone use, indicating a relevant burden in this student population.
- Higher problematic smartphone use was associated with poorer physical, psychological, and environmental quality of life, suggesting that digital behavior may affect multiple dimensions of student well-being.

**Public health implications—What are the key implications or messages for practitioners, policy makers and/or researchers in public health?**

- Nursing schools and student-support services should consider integrating digital well-being, sleep hygiene, self-regulation, and healthy smartphone-use strategies into student health promotion programs.
- Future research should examine the mechanisms linking problematic smartphone use with quality of life, especially stress, sleep, academic workload, and environmental pressures during nursing education.

**Abstract:**

Background: Problematic smartphone use may threaten student well-being, especially among nursing students who rely on smartphones for academic and clinical activities. This study estimated potential problematic smartphone use among Greek nursing students, examined its association with quality of life (QoL), and explored whether contextual factors modified these associations. Methods: A cross-sectional study was conducted among 331 nursing students in Greece from September to November 2025. Participants completed an anonymous online questionnaire including sociodemographic data, the Smartphone Addiction Scale–Short Version (SAS-SV), and the World Health Organization Quality of Life–BREF (WHOQOL-BREF). Results: The mean SAS-SV score was 29.30 ± 9.69, and 18.9% of students screened positive for potential problematic smartphone use. Mean overall QoL and general health satisfaction were 3.80 ± 0.78 and 3.97 ± 0.88, respectively. Higher SAS-SV scores were associated with lower physical, psychological, and environmental QoL, but not with social QoL. Years of study moderated only the association with environmental QoL. Conclusions: Problematic smartphone use was associated with poorer physical, psychological, and environmental QoL among Greek nursing students. These domain-specific findings extend existing evidence and support integrating digital well-being, self-regulation, and sleep-hygiene strategies into nursing education and student-support services.

## 1. Introduction

Over the past decade, smartphones have become an essential element of modern daily routine, shaping communication, social interaction, educational processes, and even healthcare delivery [1]. By combining functions traditionally associated with mobile phones and computers, smartphones offer multiple features including internet access, communication, web browsing, social media engagement, multimedia display, and educational applications. This versatility has contributed to the widespread adoption of smartphones across different contexts [2,3,4].

Smartphone use is particularly common among adolescents, young adults, and university students, including those in health-related fields such as nursing [5]. Among nursing students, smartphone use is deeply embedded in daily routines, supporting communication with peers and family, facilitating rapid access to clinical and educational resources, and enabling engagement with learning activities and social media [1]. In parallel, healthcare education and institutions have increasingly integrated smart technologies to support care quality, a trend accelerated during and after the COVID-19 pandemic [6]. Likewise, universities have expanded distance-learning platforms and digital teaching approaches, further reinforcing reliance on smartphone use among university students [7]. Collectively, this growing dependence on smartphones may have important implications for students’ physical health, welfare, quality of life (QoL), and academic progress [8].

The present study is conceptually guided by Compensatory Internet Use Theory, which proposes that problematic digital behaviors may develop when individuals use online activities to cope with stress, emotional distress, loneliness, or unmet offline needs [9]. Although the theory was originally developed in relation to internet use, it is relevant to smartphone use because smartphones function as continuous and portable gateways to internet-based communication, social media, entertainment, information-seeking, and academic resources. From this perspective, the smartphone itself is not necessarily the object of addiction; rather, problematic use may reflect difficulty regulating the activities and emotional functions accessed through the device. This framework is particularly relevant for nursing students, who may rely on smartphones for academic, clinical, and social purposes while also experiencing academic workload, clinical exposure, transition-related stress, and pressures related to professional preparation.

Despite the benefits of smartphone use, excessive and uncontrolled use, commonly described as problematic smartphone use, has been reported among nursing students [10]. In the present study, problematic smartphone use is used as the primary construct and refers to dysregulated smartphone engagement associated with difficulty controlling use, functional interference, or distress; it is not used as a clinical diagnosis. Related terms appear in the literature but are not fully interchangeable. “Smartphone addiction” is often used to describe addiction-like symptoms, although its status as a distinct clinical disorder remains debated [11]. “Smartphone dependency” usually denotes reliance on the device in everyday life, whereas “nomophobia” refers specifically to anxiety or distress when a person is without access to a mobile phone [12]. Accordingly, the present manuscript uses “problematic smartphone use” as the main term, while retaining the terminology used in cited studies when describing their findings [13,14].

Smartphone use is common in both classroom and clinical practice, with nearly half of nursing students reporting use during clinical placements and a similar proportion acknowledging distraction related to smartphones [15]. Some evidence also indicates that female undergraduate students may exhibit elevated levels of smartphone dependency and nomophobia compared to males in some populations [14]. International evidence indicates that problematic smartphone use is a widespread concern among nursing students, although reported prevalence estimates vary substantially across countries. This heterogeneity likely reflects differences in measurement instruments, cut-off scores, terminology, sampling strategies, and cultural or educational contexts; therefore, cross-country comparisons should be interpreted as study-specific screening estimates rather than as directly comparable rates of a clinical disorder. For example, studies have reported estimates of 23.1% in India [16], 34.9% in Morocco, and 10.41% in China, whereas European studies have also reported heterogeneous rates among nursing students in France, Spain, and Portugal [8,17,18]. In Greece, problematic smartphone use among nursing students has also been documented, with a prior study reporting a prevalence estimate of 16.6% [19]. Despite methodological variability, these findings collectively suggest that problematic smartphone use is a relevant issue in nursing education internationally.

Overall, problematic smartphone use among nursing students has been associated with poorer physical and psychological well-being, sleep difficulties, reduced academic engagement, and distraction during learning or clinical activities [11,15,16,17].

QoL is a subjective and multidimensional construct that includes physical health, psychological well-being, social relationships, and environmental conditions. According to the WHO, QoL refers to individuals’ perceptions of their position in life within their cultural and value context and in relation to their goals, expectations, standards, and concerns [20]. Previous studies and synthesized evidence suggest that higher levels of problematic smartphone use are associated with poorer QoL indicators among university and nursing students [13,21,22]. These findings support the need to examine whether problematic smartphone use is related to specific QoL domains rather than only to overall well-being.

In addition to problematic smartphone use, students’ QoL may also be shaped by sociodemographic and lifestyle-related factors. Age and gender were included as basic demographic covariates because they may be associated with differences in perceived health, psychological well-being, lifestyle patterns, and student experience. Smoking status was included as a health-related lifestyle covariate that may be linked to physical well-being and broader QoL perceptions. Living arrangement, particularly, may influence social support, as students’ place of residence can determine their proximity to family members, peers, and other informal support networks. Students residing away from family or in dormitory settings may experience additional adjustment-related stress [23], whereas positive family interaction and social support may promote healthier lifestyles and better well-being [24,25]. Year of study was included because it may reflect stage-specific differences in academic demands, clinical exposure, and transition-related stress, as well as varying support needs throughout nursing education. Final-year nursing students may experience anxiety related to future employment, clinical training, and professional preparation [26], whereas first-year students may require greater support during adjustment to higher education [27]. These variables were therefore treated as a priori contextual covariates rather than selected on the basis of statistical significance.

Within the framework of Compensatory Internet Use Theory, problematic smartphone use may be associated with QoL through domain-specific pathways. In the physical domain, it may disturb sleep, reduce physical activity, and contribute to fatigue and somatic or musculoskeletal discomfort [28,29,30,31]. In the psychological domain, it may be linked to elevated stress, anxiety, depressive symptoms, and impaired self-regulation [32,33]. Regarding the social domain, excessive use may interfere with face-to-face interaction or substitute for offline support [34]. In the environmental domain, problematic use may impair concentration, time management, and academic functioning, while reducing students’ perceived control over daily responsibilities and available resources [35,36]. These pathways support the examination of QoL as a multidimensional outcome and suggest that the association with problematic smartphone use may vary across domains rather than appear as a uniform effect.

Evidence directly examining problematic smartphone use in relation to domain-specific QoL among Greek nursing students remains limited. Existing studies have often focused on overall well-being or single outcomes, whereas less attention has been given to whether associations differ across physical, psychological, social, and environmental QoL, or whether context-related factors in nursing education modify these associations. Therefore, this study examined potential problematic smartphone use among nursing students in Greece and its associations with WHOQOL-BREF domains, while accounting for key sociodemographic and student-life covariates. We hypothesized that higher SAS-SV scores would be associated with lower physical, psychological, and environmental QoL. The association with social QoL was examined more exploratorily, because smartphone use may both interfere with face-to-face interaction and provide access to social connection. In addition, living arrangement and year of study were explored as theoretically informed contextual moderators.

## 2. Materials and Methods

### 2.1. Sample and Data Collection

An online convenience sample of 331 undergraduate nursing students in Greece was recruited for this cross-sectional study from September 2025 to November 2025. Recruitment was carried out through digital invitations distributed via university student platforms and social media, including official university forums, student associations, and official institutional online platforms, to expand outreach and diversity of participants. The invitation targeted undergraduate nursing students from first to final year of study. Although the recruitment strategy used multiple institutional and student communication channels, institution-specific response counts were not retained. Therefore, the sample should be interpreted as an online convenience sample of undergraduate nursing students recruited through multiple communication channels, rather than as an institution-stratified, multi-site, or nationally representative sample. Eligible participants were undergraduate nursing students enrolled in a Greek nursing department, from first to final year of study, who were willing to participate and provided informed consent. Students who were not enrolled in undergraduate nursing studies, postgraduate students, and questionnaires with incomplete consent or unusable data were excluded. Data collection was conducted using a structured electronic questionnaire developed and administered through Google Forms. The electronic form was configured to require completion of the key study variables before submission, which minimized item-level missing data. The introductory section of the form included an informed consent form describing the study aims, voluntary enrollment, confidentiality assurances, and contact details of the first author. Completion time for the questionnaire was approximately 10–15 min. Participants were instructed to complete the questionnaire only once. Because the questionnaire was anonymous, duplicate responses could not be fully ruled out; however, the dataset was screened for exact duplicate records and implausible response patterns before analysis. Although all variables were self-reported, measurement error was reduced by using validated instruments with established scoring procedures and by administering the same standardized questionnaire to all participants.

### 2.2. Sampling Technique

The required sample size was estimated using the single-proportion formula: n = Z^2^p(1 − p)/d^2^, where n denotes the required sample size, Z is the standard normal value corresponding to the selected confidence level, p is the expected proportion, and d is the desired margin of error The calculation assumed a 95% confidence level (Z = 1.96), a 5% margin of error (d = 0.05), and an expected proportion of 0.235 for potential problematic smartphone use based on previous nursing-student literature. Because an official, contemporaneous source confirming the exact total number of eligible undergraduate nursing students across Greek nursing departments was not available for this study, finite population correction was not applied. The calculation produced a minimum required sample of approximately 277 participants. The final sample included 331 students, exceeding the minimum recommended sample size and improving statistical precision. This calculation was designed to estimate prevalence with adequate precision; no separate a priori power analysis was conducted specifically for the regression or moderation models. Therefore, moderation findings, particularly interaction effects, should be interpreted as exploratory.

### 2.3. Measurements

The survey tool comprised three parts: (a) socio-demographic characteristics, (b) the Smartphone Addiction Scale-Short Version (SAS-SV), and (c) the World Health Organization QoL—BREF instrument (WHOQOL-BREF).

#### 2.3.1. Socio-Demographic Characteristics

The socio-demographic characteristics questionnaire, developed for the study, included questions about gender, age, year of study, smoking status and living arrangement. Living arrangement was measured as a binary variable, distinguishing students living alone from those living with a partner or family. This categorization was intended to approximate the presence of household-based social support. However, it does not capture other common student arrangements, such as living with peers, in student housing or dormitories, or in other shared arrangements. Therefore, findings related to living arrangement should be interpreted as broad indicators of living context rather than detailed comparisons between housing types.

#### 2.3.2. The Smartphone Addiction Scale-Short Version (SAS-SV)

The Smartphone Addiction Scale-Short Version (SAS-SV), developed by Kwon et al. [37] is a validated self-report instrument designed to assess problematic or addictive smartphone use, particularly among adolescents and young adults. It is the abbreviated form of the original Smartphone Addiction Scale (SAS) and consists of 10 items rated on a 6-point Likert scale ranging from 1 “strongly disagree” to 6 “strongly agree”. The SAS-SV provides a total screening score reflecting problematic smartphone-use symptoms, such as loss of control, sleep disturbance, functional interference, and emotional dependence; however, only the total score was used in the present analyses. In the original SAS-SV validation study, the instrument demonstrated excellent internal consistency (Cronbach’s α > 0.90). In the present study, Cronbach’s α was 0.862. The SAS-SV total score ranges from 10 to 60, with higher scores indicating greater problematic smartphone use. Following Kwon et al. [37], sex-specific SAS-SV cut-off values of 31 for males and 33 for females were applied as screening thresholds for potential problematic smartphone use. Because these cut-off scores were originally proposed in an adolescent SAS-SV validation sample and have not been specifically validated as diagnostic thresholds for Greek nursing students, they were used only to identify students who screened positive for potential problematic smartphone use. They should therefore not be interpreted as clinical diagnostic criteria. The SAS-SV was translated and culturally adapted in line with the guidelines by Beaton et al. [38] for cross-cultural adaptation of self-reported measures. The process included two independent forward translations, reconciliation into a single harmonized version, blind back-translation, and review by an expert panel to ensure semantic, idiomatic and conceptual equivalence. The translation panel included two bilingual translators with experience in health-related questionnaire translation, and the expert panel consisted of nursing academics and methodological experts. Cognitive debriefing interviews with 10 participants (approximately 5–10 min each) were conducted to assess clarity and cultural appropriateness, with no reported difficulties in understanding the items. Participants did not report difficulties in understanding the items, and no substantive item-content changes were required following this process. Given that the present study focused on the association between SAS-SV scores and QoL rather than on psychometric validation, no separate factor analysis of the adapted SAS-SV was conducted.

#### 2.3.3. The World Health Organization QoL—BREF (WHOQOL-BREF)

QoL was examined using the World Health Organization QoL—BREF (WHOQOL-BREF), a 26-item self-report instrument derived from the original WHOQOL-100 and developed cross-culturally by the WHOQOL Group [39]. The WHOQOL-BREF generates four domain scores: Physical Health (7 items), Psychological Health (6 items), Social Relationships (3 items), and Environment (8 items), in addition to two single items that assess overall QoL and general health. Items are rated on 5-point Likert-type scales reflecting intensity, frequency, satisfaction. Domain scores are calculated according to WHO guidelines and transformed to a 0–20 scale, with higher scores indicating better perceived QoL. The Greek version of the WHOQOL-BREF has demonstrated satisfactory internal consistency, construct validity, and discriminant validity in both clinical and general populations, supporting its use in Greek-speaking samples [40]. In the present sample, internal consistency for the WHOQOL-BREF domains ranged from borderline to acceptable, with Cronbach’s α values ranging from 0.666 to 0.732.

### 2.4. Ethical Considerations

The present study followed established ethical standards, protection of participants’ rights, privacy, and confidentiality throughout all stages of the research. All participants were notified that participation to this study was entirely voluntary, and the time required to complete the survey. They were also informed of their right to withdraw from the study at any time without any penalty. The study was conducted in accordance with the Declaration of Helsinki and received ethical approval from the Ethics Committee of Democritus University of Thrace (Approval 55705/475).

### 2.5. Statistical Analysis

Statistical analyses were performed using IBM SPSS Statistics for Windows, version 27.0 (IBM Corp., Armonk, NY, USA). Data were screened for completeness, out-of-range values, exact duplicate records, and implausible response patterns before analysis. No missing values were identified for the variables included in the present analyses; therefore, no imputation was performed. The final analytic sample consisted of 331 participants, and all 331 participants were included in the descriptive analyses, bivariate analyses, adjusted regression models, and moderation analyses. Descriptive statistics were used to summarize sample characteristics and study variables and included means, standard deviations, frequencies, and percentages. WHOQOL-BREF raw domain scores were calculated according to WHO guidelines and linearly transformed to a 0–20 scale, with higher scores indicating better QoL. Normality of continuous variables was assessed using the Kolmogorov–Smirnov test. As several variables were not normally distributed, non-parametric analyses were used for bivariate comparisons. Specifically, Spearman’s rho was used to examine the association between SAS-SV total score and QoL domains; Mann–Whitney U tests were used to compare QoL domain scores across binary categorical variables (gender, living arrangement, and smoking status); and Kruskal–Wallis H tests were used to compare QoL domain scores across year of study.

Multiple linear regression models were fitted separately for each WHOQOL-BREF domain to estimate the adjusted association between SAS-SV total score and QoL after controlling for age, gender, year of study, living arrangement, and smoking status. Gender, living arrangement, smoking status, and year of study were entered as categorical predictors using dummy coding. Male gender, living alone, non-smoking status, and first year of study were used as the reference categories. Although non-parametric tests were used for bivariate comparisons because several variables were not normally distributed, linear regression was considered appropriate for the adjusted analyses because the dependent variables were continuous domain scores and regression assumptions were evaluated at the residual level. Residual normality was assessed using histograms and normal probability plots of standardized residuals. Linearity and homoscedasticity were examined using scatterplots of standardized predicted values against standardized residuals. Multicollinearity was assessed using variance inflation factors, and influential observations were evaluated using Cook’s distance. No evidence of serious multicollinearity or highly influential observations was identified. For each regression model, unstandardized coefficients, standard errors, 95% confidence intervals, standardized coefficients, *p*-values, R^2^, and adjusted R^2^ were reported. Moderation analyses were conducted in IBM SPSS Statistics, version 25, using the standard Linear Regression procedure. No external macro was used. These analyses examined whether the association between SAS-SV total score and each WHOQOL-BREF domain differed according to year of study or living arrangement. The SAS-SV total score was mean-centered before creating product interaction terms. Year of study was dummy-coded using first year as the reference category, and living arrangement was dummy-coded using living alone as the reference category. In total, eight moderation tests were conducted, corresponding to two contextual moderators, year of study and living arrangement, across the four WHOQOL-BREF domains. The year-of-study interaction was tested as a block of dummy-coded interaction terms. Because these moderation analyses were exploratory and theory-informed rather than confirmatory, no formal correction for multiple testing was applied. Therefore, statistically significant interaction findings were interpreted cautiously and considered preliminary. Subgroup-specific slopes were obtained by running separate linear regression models within each academic-year subgroup, with environmental QoL as the dependent variable and SAS-SV total score as the independent variable. Statistical significance was set at *p* < 0.05.

### 2.6. Use of Generative AI-Assisted Tool

During manuscript preparation, LeapSpace AI was used as an AI-assisted research-support tool to facilitate preliminary literature exploration, identify potentially relevant scholarly sources, and support the development of ideas for further manual review. The tool was not used for data collection, data analysis, statistical testing, generation of results, preparation of tables, or interpretation of the study findings. All AI-assisted outputs, including suggested literature and conceptual prompts, were independently reviewed, verified against original sources, critically evaluated, and edited by the authors. The authors retained full control over the content of the manuscript and take full responsibility for the accuracy, integrity, and final version of the work.

## 3. Results

A total of 331 nursing students participated in the study. Most participants were females (74.3%), in their 1st year of studies (46.5%), were living on their own (62.8%), and were nonsmokers (67.6%). The mean age of the sample was 23.1 ± 7.79. The sample included a high proportion of first-year students and a broad age distribution, suggesting that the findings may reflect the experiences of both traditional-age and older nursing students. The demographic characteristics of the participants are presented in Table 1.

Descriptive statistics and Cronbach’s α for the scales (SAS-SV and WHOQOL-BREF) are presented in Table 2. On the two WHOQOL-BREF single items scored from 1 to 5, mean overall QoL was 3.80 ± 0.78 and mean general health satisfaction was 3.97 ± 0.88, indicating generally favorable self-perceived well-being. The SAS-SV total score was 29.30 ± 9.69. Using the sex-specific SAS-SV screening thresholds (≥31 for males and ≥33 for females), 111/331 students (33.5%) screened positive for potential problematic smartphone use. Specifically, 32/85 male students (37.6%) and 79/246 female students (32.1%) exceeded the screening threshold. Regarding QoL domains, the physical health domain had the highest mean score (15.36 ± 2.37), followed by the social relationships domain (15.10 ± 3.42) whereas the environmental health domain had the lowest score (14.03 ± 2.34). The Cronbach’s α coefficient for the SAS-SV was 0.862, indicating strong internal consistency. For the WHOQOL-BREF, Cronbach’s α coefficients were 0.725 for physical health, 0.705 for psychological health, 0.666 for social relationships, and 0.732 for environment, indicating borderline to acceptable internal consistency across domains. The social relationships domain showed the lowest internal consistency; therefore, findings involving this domain should be interpreted with some caution. 

Table 3 displays bivariate analyses between demographic characteristics, the SAS-SV and the WHOQOL-BREF domains. Students’ physical, psychological and environmental health were found to be associated negatively with the total SAS-SV score. Male students had higher median psychological health and environmental QoL scores than female students (*p* = 0.012 and *p* = 0.021, respectively), while no statistically significant gender differences were observed for physical health or social relationships. Students living with a partner or family had higher median social relationships scores than those living alone (*p* < 0.001), whereas no statistically significant differences were observed for the physical, psychological, or environmental domains. Regarding the magnitude of the associations, the correlation between SAS-SV total score and physical health was moderate and negative, whereas the correlations with psychological health, social relationships, and environmental QoL were small and negative. These findings suggest that problematic smartphone use was more clearly related to physical QoL than to the other QoL domains at the bivariate level.

In adjusted linear regression models, higher SAS-SV total scores were independently associated with lower physical health (B = −0.087, 95% CI −0.121 to −0.052, β = −0.352, *p* < 0.001), psychological health (B = −0.055, 95% CI −0.091 to −0.019, β = −0.226, *p* = 0.003), and environmental QoL (B = −0.069, 95% CI −0.104 to −0.035, β = −0.286, *p* < 0.001), but not with social relationships (B = −0.036, 95% CI −0.087 to 0.014, β = −0.104, *p* = 0.159). Living arrangement was independently associated with social relationships (B = 1.608, *p* < 0.001), and gender was independently associated with environmental QoL (B = −1.030, *p* = 0.006). Standardized coefficients indicated that the strongest association between problematic smartphone use and QoL was observed for physical health, followed by environment and psychological health. The regression models explained a modest proportion of variance across QoL domains, with R^2^ values ranging from 0.077 to 0.155. This indicates that problematic smartphone use and the included covariates accounted for only part of the variability in QoL, and that other unmeasured factors, such as stress, anxiety, depression, sleep quality, academic workload, physical activity, and social support, may also be important.

In moderation analyses, a significant interaction was observed only for the environmental domain. The addition of the study year × SAS-SV total interaction block significantly improved the model fit (ΔR^2^ = 0.034, ΔF(3, 319) = 4.362, *p* = 0.005), whereas no significant interactions were found for physical, psychological, or social QoL. Post hoc interpretation indicated that the negative association between SAS-SV total score and environmental QoL was most pronounced among fourth-year students, whereas the association was weaker among earlier academic years. However, because the number of students was unevenly distributed across years of study, this interaction should be interpreted cautiously and considered exploratory (Table 4). To clarify the direction and strength of this interaction, subgroup-specific estimates were examined for the association between SAS-SV total score and environmental QoL within each year of study. These subgroup-specific estimates are presented in Supplementary Table S2.

## 4. Discussion

Problematic smartphone use represents a substantial health-related challenge among undergraduate students, due to its potential harmful effects on mental health, academic achievement, and daily routine [41]. In this cross-sectional sample of nursing students, greater levels of problematic smartphone use were associated with lower QoL across multiple domains. In adjusted analyses controlling for key sociodemographic characteristics, problematic smartphone use remained negatively related to physical health, psychological health, and environmental QoL domains, whereas the association with the social relationship domain was not statistically significant after covariate adjustment. Taken together, these findings suggest that the association between problematic smartphone use and QoL was domain-specific rather than uniform across all QoL dimensions.

The magnitude of these associations should be interpreted carefully. Although several associations reached statistical significance, the regression coefficients and explained variance values indicated modest effects. The adjusted models explained 7.7% to 15.5% of the variance in QoL domains, showing that problematic smartphone use was one correlate of students’ QoL rather than a dominant determinant. In practical terms, a 10-point higher SAS-SV score corresponded to approximately 0.87 points lower physical QoL, 0.55 points lower psychological QoL, and 0.69 points lower environmental QoL on the 0–20 WHOQOL-BREF scale. Therefore, other unmeasured factors, such as stress, sleep quality, academic workload, physical activity, social support, anxiety, and depressive symptoms, may also contribute substantially to students’ QoL. The absence of an independent association with social QoL may reflect the more complex role of smartphones in students’ social lives; however, this interpretation remains hypothetical because the present study did not directly assess the quality of online and offline social interactions. Although excessive smartphone use may interfere with face-to-face interaction, smartphones may also help students maintain communication with family, peers, and support networks. In the present study, social QoL appeared to be more strongly related to living arrangement than to SAS-SV total score, suggesting that household-based social support may be more relevant to perceived social relationships than problematic smartphone use alone. Therefore, problematic smartphone use should be interpreted as one relevant correlate of QoL, rather than as a dominant explanation for students’ well-being outcomes.

The overall scores of the physical health domain indicated a generally positive perception of physical well-being. Nevertheless, nearly one-fifth of the study’s sample met the screening criteria for potential problematic smartphone use, and higher SAS-SV scores were independently associated with lower physical QoL. This was the strongest adjusted association observed in the study, suggesting that problematic smartphone use may be particularly relevant to aspects of QoL involving sleep, energy, discomfort, daily functioning, and capacity to perform usual activities. This interpretation is consistent with previous evidence linking excessive smartphone use with poorer sleep quality, fatigue, sedentary behavior, reduced physical activity, postural discomfort, and musculoskeletal symptoms [42,43,44,45,46,47]. Rather than reflecting screen time alone, the association with physical QoL may therefore represent the broader burden of dysregulated smartphone use on rest, bodily comfort, and daily functioning.

A similar but weaker pattern was observed for psychological QoL. Higher SAS-SV scores were associated with poorer psychological QoL after adjustment for demographic and lifestyle factors, although the explained variance of the model was modest. This suggests that problematic smartphone use is one relevant correlate of psychological well-being, but not its only determinant. From the perspective of Compensatory Internet Use Theory, students experiencing stress, emotional strain, loneliness, or reduced self-regulation may use smartphones for immediate relief, distraction, reassurance, or social connection [9]. When this pattern becomes difficult to regulate, it may coexist with poorer psychological well-being. This finding is compatible with previous evidence linking problematic smartphone use with psychological distress, anxiety, depressive symptoms, sleep difficulties, reduced resilience, lower well-being, and related adverse psychological outcomes [48,49,50,51,52,53,54]. However, the present findings cannot determine the direction of this relationship. It is possible that problematic smartphone use contributes to poorer psychological QoL, suggesting that students with poorer psychological well-being are more vulnerable to dysregulated smartphone use, or that both are influenced by other factors such as academic stress, sleep disruption, or emotional strain.

Significant gender differences were observed, with male students demonstrating higher psychological and environmental QoL than female students, while no significant differences were identified in the other domains. Notably, the gender disparity in environmental QoL remained significant after adjustment for demographic characteristics, lifestyle factors, and problematic smartphone use, highlighting that female students continued to report lower perceived environmental conditions independent of these variables. Although these differences should be interpreted with caution, they suggest that QoL and digital behavior may be influenced by broader academic and sociocultural conditions that vary across student groups. This observation aligns with Paudel et al. [55] who reported higher QoL among male students across all domains, including psychological and environmental aspects, than female students. Conversely, Ali et al. [56], found no notable gender-based variances in any of QoL domains including psychological and environmental domains.

In the present study, gender differences were observed for psychological and environmental QoL, but a formal comparison of SAS-SV scores by gender was not reported. Therefore, these QoL differences should not be attributed to higher problematic smartphone use among female students. The persistence of the gender effect for environmental QoL after adjustment suggests that broader academic, social, or contextual factors may play a role in shaping these differences. Previous evidence on gender differences in smartphone-related behaviors is mixed. Roque-Hernández et al. [57] reported a significant association between gender and daily smartphone-use duration, with female students showing higher usage patterns than male students. In contrast, Khan et al. [58], observed no significant gender-based differences in levels of smartphone-use levels, although males demonstrated slightly higher scores than females. Collectively, these findings highlight the importance of interpreting gender differences in student well-being within a broader contextual framework rather than attributing them to inherent gender characteristics. They also support the need for gender-sensitive but not gender-stereotyped approaches when addressing digital behavior and QoL outcomes among nursing students.

Living arrangement emerged as a significant and independent factor of social QoL in the current study. Students residing with a partner or family demonstrated higher scores in the social relationship domain compared with those living alone, and this pattern was consistent across both bivariate and multivariate analyses. This association remained significant after controlling for demographic characteristics, lifestyle factors, and problematic smartphone use, indicating that living arrangement independently contributes to students’ perceived social well-being. This result aligns with da-Silva-Domingues et al. [29], who reported a positive moderate association between family dynamics and social support, suggesting that living context serves as an important factor in shaping university students’ social experiences. In contrast, Bošković et al. [59] found no statistically significant differences in the social relationships domain between students residing as tenants and those living with family. However, the same study reported that students living as tenants exhibited an elevated rate of interpersonal sensitivity, highlighting greater challenges in social interactions and relationships. The absence of significant relation between living arrangements and problematic smartphone use highlights that the beneficial effect of living with a partner or family on social QoL operates independently of smartphone use levels. Given that the majority of participants lived alone, this finding highlights the importance of living context as a key factor in students’ social experiences, with cohabitation with a partner or family having a stronger influence on social QoL than smartphone use. This observation is supported by Ahn et al. [60] who reported that students spending greater time in a supportive home environment were less likely to develop problematic smartphone use patterns.

The environmental domain of the QoL reflects broader living conditions and access to essential resources, including safety, financial resources, access to health and social services, leisure occasions, transport, and the home environment [61]. Problematic smartphone use was associated with lower environmental QoL, and this association was moderated by year of study. The subgroup-specific estimates suggested that the negative association was strongest among fourth-year students, while it was weaker or non-significant in earlier years. However, this finding should be interpreted cautiously because the moderation effect was limited to the environmental domain and the distribution of participants across academic years was uneven. Therefore, the result should be viewed as exploratory and domain-specific rather than as definitive evidence of a consistent year-of-study effect. This pattern aligns with Demirkan et al. [53] who reported a significant association between year of study and problematic smartphone use, suggesting that smartphone-use patterns may vary across academic levels. In contrast, Naeem et al. [62] reported no statistically significant relationship between year of study and the environmental domain of QoL. One hypothesis is that environmental QoL may be more sensitive to cumulative demands in later stages of nursing education, including clinical placements, academic workload, transition-related concerns, employment preparation, commuting demands, and time-management pressures. However, these factors were not directly measured in the present study. Therefore, this explanation requires direct testing in future research and should not be interpreted as evidence of an identified mechanism. Future studies should include direct measures of clinical workload, academic pressure, employment-related concerns, time management, and perceived environmental resources to clarify the mechanisms underlying this domain-specific moderation effect.

Overall, these findings support a domain-specific interpretation of QoL among nursing students. Digital well-being initiatives in nursing education may therefore benefit from addressing physical and psychological well-being, while also considering environmental pressures during later stages of training.

### 4.1. Study Limitations

Several limitations should be acknowledged when interpreting these findings. First, the cross-sectional design does not allow for causal or temporal inference; therefore, the observed associations cannot determine whether problematic smartphone use leads to poorer QoL, whether poorer QoL increases susceptibility to problematic smartphone use, or whether both are influenced by unmeasured confounding variables. In addition, the SAS-SV cut-off scores used to estimate potential problematic smartphone use were derived from the original adolescent SAS-SV validation sample and have not been specifically validated as diagnostic thresholds for Greek nursing students. Therefore, the prevalence estimate should be interpreted as a screening indicator rather than as evidence of clinical diagnosis. Although the adapted SAS-SV demonstrated good internal consistency in the present sample, no exploratory or confirmatory factor analysis was conducted. Therefore, the present study cannot confirm the structural validity, dimensional structure, or measurement equivalence of the adapted Greek SAS-SV among nursing students. The SAS-SV findings should consequently be interpreted at the total-score screening level, and future studies should examine its factor structure and measurement invariance in Greek nursing-student populations. Moreover, potentially important confounding variables, such as anxiety, depression, perceived stress, sleep quality, academic workload, physical activity, and social support, were not measured. The absence of these variables may have limited the ability to fully explain the associations between problematic smartphone use and QoL. Second, the convenience-based online recruitment strategy may have contributed to selection bias and restricted the generalizability of the findings. It is possible that students who were more digitally engaged, more interested in the topic, or more willing to participate in and complete online questionnaires were disproportionately represented in the sample. In addition, as institution-specific response counts were not retained, it was not possible to assess departmental or institutional representativeness. Consequently, the results should be interpreted as originating from an online convenience sample recruited through multiple institutional and student communication channels, rather than from an institution-stratified, multi-site, or nationally representative sample. Third, all measures were self-reported and may be affected by recall bias, shared-method variance, and social desirability, which could have influenced both the prevalence estimates and the observed associations. Fourth, the unequal distribution of participants across academic years, with first-year students representing nearly half of the sample, may have limited subgroup comparability and influenced the stability and interpretability of the moderation analyses. In particular, the interaction observed for the environmental domain should be interpreted with caution, as it may partially reflect uneven group sizes rather than a robust stage-of-training effect. In addition, living arrangement was measured using a simplified binary classification, and the social relationships domain demonstrated comparatively lower internal consistency. Both factors may have reduced sensitivity to detect more nuanced associations. Finally, the social relationships domain of the WHOQOL-BREF demonstrated relatively low internal consistency in the present sample, which may have reduced measurement precision and limited the ability to detect associations involving social QoL.

### 4.2. Implications for Future Research

Future research should move beyond identifying overall associations and instead focus more directly on the underlying mechanisms linking problematic smartphone use to specific QoL domains in nursing students. The interaction observed for the environmental domain should be interpreted with caution, as moderation was not evident across the other QoL domains and may also have been affected by the unequal distribution of participants across academic years. Accordingly, future studies should investigate whether factors such as clinical workload, commuting demands, financial strain, difficulties in time management, academic pressure, and reduced perceived control help explain or amplify this association, particularly in later stages of training.

In addition, because problematic smartphone use was associated with physical and psychological QoL, but not independently with social QoL, further research is required to clarify whether distinct pathways operate across these domains. For example, sleep disruption and fatigue may be more closely linked to physical well-being, while emotional strain and stress may be more strongly associated with psychological well-being. In contrast, the effect on social functioning may be more complex, potentially involving mixed or compensatory effects. Studies using more balanced samples across institutions and academic years are also needed to determine whether the observed moderation effect in environmental QoL reflects a stable pattern related to stage of education or a sample-specific subgroup imbalance.

## 5. Conclusions

In conclusion, problematic smartphone use was associated with lower physical, psychological, and environmental QoL among nursing students, while no independent association was observed with social QoL after adjustment. These outcomes suggest that the relationship between problematic smartphone use and well-being may differ across different dimensions of QoL. The study also suggests that contextual factors may be relevant in different ways depending on the domain considered: living arrangement was independently associated with social QoL, whereas moderation by year of study was observed only for environmental QoL. The moderation finding for year of study should be interpreted as preliminary and domain-specific, and it requires confirmation in longitudinal studies with more balanced samples across academic years. Overall, these results expand the current literature by showing that problematic smartphone use and contextual characteristics are associated with student well-being in a domain-specific rather than uniform manner. Practically, the findings support the integration of digital well-being into nursing student support initiatives, with attention to physical and psychological well-being and to the environmental challenges during nursing education. From a research perspective, the findings indicate the need for longitudinal and mechanism-oriented studies examining how factors such as academic demands, stress, sleep, and day-to-day self-management relate to the association between problematic smartphone use and QoL.

## Figures and Tables

**Table 1:** Socio-demographic characteristics of the sample.

Variables	n (%)
Gender	
Male	85 (25.7%)
Female	246 (74.3%)
Age ^a^	23.1 (7.79)
Living arrangement	
Live alone	208 (62.8%)
Live with my partner/family	123 (37.2%)
Smoking status	
Yes	107 (32.4%)
No	224 (67.6%)
Year of study	
1st	154 (46.5%)
2nd	36 (10.9%)
3rd	42 (12.7%)
4th	99 (29.9%)

^a^ mean (SD).

**Table 2:** Descriptive statistics and Cronbach’s α for the SAS-SV and WHOQOL-BREF.

Scale	Mean ± SD	Cronbach’s α
SAS-SV	29.30 ± 9.69	0.862
Physical health	15.36 ± 2.37	0.725
Psychological health	14.30 ± 2.34	0.705
Social relationships	15.10 ± 3.42	0.666
Environment	14.03 ± 2.34	0.732

**Table 3:** Bivariate analyses between demographic characteristics, the SAS-SV and the WHOQOL-BREF domains.

Variables	Physical Health		Psychological Health		Social Relationships		Environmental Health	
	Median (IQR)	*p*-Value	Median (IQR)	*p*-Value	Median (IQR)	*p*-Value	Median (IQR)	*p*-Value
Gender		0.076		0.012		0.523		0.021
Male	16.57 (3.29)		15.33 (2.67)		16.00 (6.33)		15.00 (3.25)	
Female	15.43 (2.86)		14.00 (3.33)		16.00 (4.00)		14.00 (3.00)	
Living arrangement		0.426		0.126		*p* < 0.001		0.255
Live alone	16.00 (2.86)		14.00 (2.67)		14.67 (5.33)		14.00 (3.00)	
Live with my partner/family	15.43 (3.43)		14.67 (4.00)		17.33 (4.00)		14.50 (3.50)	
Smoking status		0.066		0.699		0.510		0.995
Yes	15.43 (3.00)		14.00 (3.50)		16.00 (5.67)		14.50 (3.63)	
No	16.00 (2.86)		14.67 (2.67)		16.00 (4.00)		14.00 (3.00)	
Year of study		0.300		0.959		0.843		0.176
1st	16.57 (2.86)		14.67 (2.67)		16.00 (4.00)		14.50 (3.00)	
2nd	14.86 (3.14)		14.00 (2.50)		16.00 (5.33)		13.75 (3.25)	
3rd	16.57 (4.29)		13.33 (3.67)		16.00 (4.67)		13.00 (3.25)	
4th	15.43 (3.14)		14.67 (3.33)		16.00 (5.33)		14.00 (4.00)	
	Physical health ρ (*p*)		Psychological health ρ (*p*)		Social relationships ρ (*p*)		Environmental health ρ (*p*)	
SAS-SV	−0.401 (*p* < 0.001)		−0.228 (*p* < 0.001)		−0.138 (*p* = 0.012)		−0.247 (*p* < 0.001)	

Values for categorical variables are presented as median (interquartile range). *p*-values for gender, living arrangement, and smoking status were derived from the Mann–Whitney U test; *p*-values for year of study were derived from the Kruskal–Wallis H test. Correlations between SAS-SV total score and WHOQOL-BREF domains were calculated using Spearman’s ρ. WHOQOL-BREF domain scores were transformed to a 0–20 scale, with higher values indicating better quality of life.

**Table 4:** Association between SAS-SV total and WHOQOL-BREF domains, adjusted regressions and moderation tests.

WHOQOL-BREF Domain	Adjusted Regression R^2^	SAS-SV Total B (95% CI)	SAS-SV β	*p*-Value	Other Significant Covariates	Significant Moderation (SAS-SV Total × Subgroup
Physical health	0.155	−0.087 [−0.121, −0.052]	−0.352	<0.001	None	No (study year interaction *p* = 0.442; other subgroup interactions ns)
Psychological health	0.077	−0.055 [−0.091, −0.019]	−0.226	0.003	None	No (study year × SAS-SV total *p* = 0.813; subgroup interactions ns)
Social relationships	0.098	−0.036 [−0.087, 0.014]	−0.104	0.159	Living arrangement: +1.608, *p* < 0.001	No (study year × SAS-SV total *p* = 0.836; living arrangement × SAS-SV total *p* = 0.327)
Environment	0.137	−0.069 [−0.104, −0.035]	−0.286	<0.001	Gender: −1.030, *p* = 0.006	Yes: Study year × SAS-SV total interaction block: ΔR^2^ = 0.034, ΔF(3, 319) = 4.362, *p* = 0.005

Note. Regression models were adjusted for age, gender, living arrangement, smoking status, and year of study. Unstandardized coefficients (B), 95% confidence intervals (CI), standardized coefficients (β), *p*-values, and R^2^ are shown for SAS-SV total score. WHOQOL-BREF domain scores were transformed to a 0–20 scale, with higher scores indicating better quality of life. Moderation analyses tested whether the association between SAS-SV total score and QoL differed across selected subgroups. ns = not significant. A significant interaction was observed only for the environmental domain. The full adjusted regression models, including all covariates, unstandardized coefficients, standard errors, 95% confidence intervals, standardized coefficients, *p*-values, R^2^, and adjusted R^2^ values, are presented in Supplementary Table S1. Subgroup-specific simple slopes for the significant environmental QoL moderation model are presented in Supplementary Table S2. The interaction-block statistics are reported using ΔR^2^ and the change-in-F statistic.

## Data Availability

The data presented in this study are available on request from the corresponding author. The data are not publicly available due to privacy and ethical restrictions.

## Supplementary Materials

The following supporting information can be downloaded at: https://www.mdpi.com/article/10.3390/ijerph23070870/s1, Supplementary Table S1: Full adjusted regression models for the association between SAS-SV total score and WHOQOL-BREF domains; Supplementary Table S2: Subgroup-specific estimates for the association between SAS-SV total score and environmental QoL by year of study.

## Abbreviations

The following abbreviations are used in this manuscript:

QoL	Quality of life
SAS	Smartphone Addiction Scale
SAS-SV	Smartphone Addiction Scale–Short Version
SD	Standard deviation
WHO	World Health Organization
WHOQOL-BREF	World Health Organization Quality of Life–BREF
