# Supplementary material for: Problematic Smartphone Use and Quality of Life Among Greek Nursing Students: A Cross-Sectional Study
Source: Int J Environ Res Public Health. 2026 Jul 3;23(7):870. doi: 10.3390/ijerph23070870 (PMC13410332; doi:10.3390/ijerph23070870)
Supplement: Supplementary file 1 [file ijerph-23-00870-s001.zip › ijerph-4375289-supplementary.pdf]

Supplementary Table S1. Full adjusted regression models for the association between SAS-SV total score and WHOQOL-BREF domains

WHOQOL-BREF domain	Predictor	B	SE	95% CI	β	p-value
Physical health	SAS-SV total	-0.087	0.018	-0.121 to -0.052	-0.352	<0.001
Physical health	Age	0.008	0.018	-0.028 to 0.043	0.026	0.667
Physical health	Female gender	-0.445	0.277	-0.989 to 0.100	-0.083	0.109
Physical health	Living with partner/family	-0.003	0.267	-0.528 to 0.522	-0.001	0.992
Physical health	Smoking	-0.568	0.607	-1.762 to 0.626	-0.114	0.350
Physical health	2nd year of study	-0.433	0.450	-1.319 to 0.452	-0.053	0.336
Physical health	3rd year of study	0.267	0.393	-0.506 to 1.040	0.038	0.497
Physical health	4th year of study	-0.201	0.314	-0.819 to 0.416	-0.040	0.522
Psychological health	SAS-SV total	-0.055	0.018	-0.091 to -0.019	-0.226	0.003
Psychological health	Age	-0.003	0.019	-0.041 to 0.035	-0.010	0.872
Psychological health	Female gender	-0.455	0.288	-1.022 to 0.111	-0.087	0.115
Psychological health	Living with partner/family	0.531	0.280	-0.021 to 1.082	0.111	0.059
Psychological health	Smoking	0.059	0.272	-0.476 to 0.594	0.012	0.829
Psychological health	2nd year of study	0.337	0.465	-0.578 to 1.253	0.042	0.469
Psychological health	3rd year of study	0.460	0.406	-0.339 to 1.260	0.068	0.258
Psychological health	4th year of study	0.264	0.332	-0.389 to 0.917	0.052	0.427
Social relationships	SAS-SV total	-0.036	0.026	-0.087 to 0.014	-0.104	0.159
Social relationships	Age	0.047	0.026	-0.005 to 0.099	0.110	0.077
Social relationships	Female gender	-0.065	0.403	-0.858 to 0.727	-0.009	0.871
Social relationships	Living with partner/family	1.608	0.388	0.845 to 2.371	0.246	<0.001
Social relationships	Smoking	-0.044	0.380	-0.792 to 0.704	-0.006	0.908
Social relationships	2nd year of study	0.809	0.655	-0.480 to 2.098	0.070	0.218
Social relationships	3rd year of study	0.179	0.572	-0.946 to 1.304	0.018	0.755
Social relationships	4th year of study	-0.860	0.457	-1.759 to 0.039	-0.119	0.061
Environment	SAS-SV total	-0.069	0.018	-0.104 to -0.035	-0.286	<0.001
Environment	Age	-0.005	0.018	-0.040 to 0.031	-0.016	0.791
Environment	Female gender	-1.030	0.372	-1.763 to -0.297	-0.198	0.006
Environment	Living with partner/family	0.620	0.328	-0.026 to 1.266	0.129	0.060

WHOQOL-BREF domain	Predictor	B	SE	95% CI	β	p-value
Environment	Smoking	0.064	0.260	-0.447 to 0.575	0.013	0.806
Environment	2nd year of study	-0.681	0.447	-1.561 to 0.200	-0.084	0.129
Environment	3rd year of study	-0.966	0.610	-2.165 to 0.233	-0.140	0.114
Environment	4th year of study	-0.542	0.312	-1.156 to 0.072	-0.108	0.084

Model fit: Physical health: R^2 = **0.155**, adjusted R^2 = **0.134**. Psychological health R^2 = **0.077**, adjusted R^2 = **0.053**. Social relationships: R^2 = **0.098**, adjusted R^2 = **0.075**. Environment: R^2 = **0.137**, adjusted R^2 = **0.115**.

Note. B = unstandardized coefficient; SE = standard error; CI = confidence interval; β = standardized coefficient; SAS-SV = Smartphone Addiction Scale–Short Version; WHOQOL-BREF = World Health Organization Quality of Life–BREF. All models were adjusted for age, gender, living arrangement, smoking status, and year of study. Reference categories were male gender, living alone, non-smoking status, and first year of study. WHOQOL-BREF domain scores were transformed to a 0–20 scale, with higher values indicating better quality of life.

Supplementary Table S2. Subgroup-specific estimates for the association between SAS-SV total score and environmental QoL by year of study

Year of study	B	SE	95% CI	p-value
1st year	-0.019	0.021	-0.061 to 0.022	0.353
2nd year	0.044	0.038	-0.031 to 0.118	0.253
3rd year	-0.102	0.028	-0.157 to -0.047	<0.001
4th year	-0.134	0.021	-0.175 to -0.092	<0.001

Note. B = unstandardized coefficient; SE = standard error; CI = confidence interval; SAS-SV = Smartphone Addiction Scale–Short Version; QoL = quality of life. Estimates represent subgroup-specific simple slopes for the association between SAS-SV total score and environmental QoL within each year of study, derived from the moderation model adjusted for age, gender, living arrangement, and smoking status. Environmental QoL scores were transformed to a 0–20 scale, with higher scores indicating better quality of life. For the environmental QoL moderation model, addition of the study year × SAS-SV total interaction block improved model fit by $\Delta R^2 = 0.034$, $\Delta F(3, 319) = 4.362$, $p = 0.005$.
